# Electronic and Structural Heterogeneity in the Diiron Center of Sulerythrin: Insights From Hybrid QM/MM Calculations

**DOI:** 10.1002/cphc.202500772

**Published:** 2026-01-25

**Authors:** Samah Moubarak, Maria Andrea Mroginski

**Affiliations:** ^1^ Institut für Chemie Sekr. PC7 Technische Universität Berlin Berlin Germany

**Keywords:** catalysis, DFT, enzyme, iron, quantum mechanical/molecular mechanics

## Abstract

Sulerythrins (SulE) are ferritin‐like proteins from obligate aerobes such as *Sulfolobus tokodaii*, forming a domain‐swapped dimer with a four‐helix‐bundle scaffold and a heterobimetallic Fe–Zn center. The diFe‐SulE variant resembles diiron carboxylate proteins and contains two bimetallic active sites coordinated by histidines, glutamates, and bridging oxo ligands. High‐resolution crystallography revealed slight differences in Fe–Fe distances and mixed‐valence states, but the precise chemical nature of the oxo species remains unclear. To clarify the electronic and structural properties of diFe‐SulE, we performed hybrid quantum mechanical/molecular mechanics (QM/MM) calculations on models varying in protonation, dioxo ligands, and iron redox states of the active site. Our results reveal at least three electronic states for diFe‐SulE: (i) a diferrous center with an end‐on di‐μ‐hydroperoxo ligand; (ii) a diferric center with hydroxo ligands interacting with protonated Glu95; and (iii) a diferrous center bridged by a di‐μ‐peroxo ligand, also interacting with protonated Glu95. These states are consistent with the structural heterogeneity observed experimentally. Overall, the hybrid QM/MM approach refines the crystallographic models and offers subatomic‐level insight into the electronic structure and reactivity of the SulE diiron center, deepening our understanding of nonheme diiron enzymes.

## Graphical abstract

QM/MM modeling of Sulerythrin (SulE) identifies three electronic states of its diiron center, differing in protonation and bridging ligands. The results reconcile crystallographic heterogeneity and reveal how subtle electronic and structural changes govern non‐heme diiron enzyme reactivity.

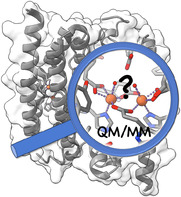

## 1 Introduction

**FIGURE 1:**
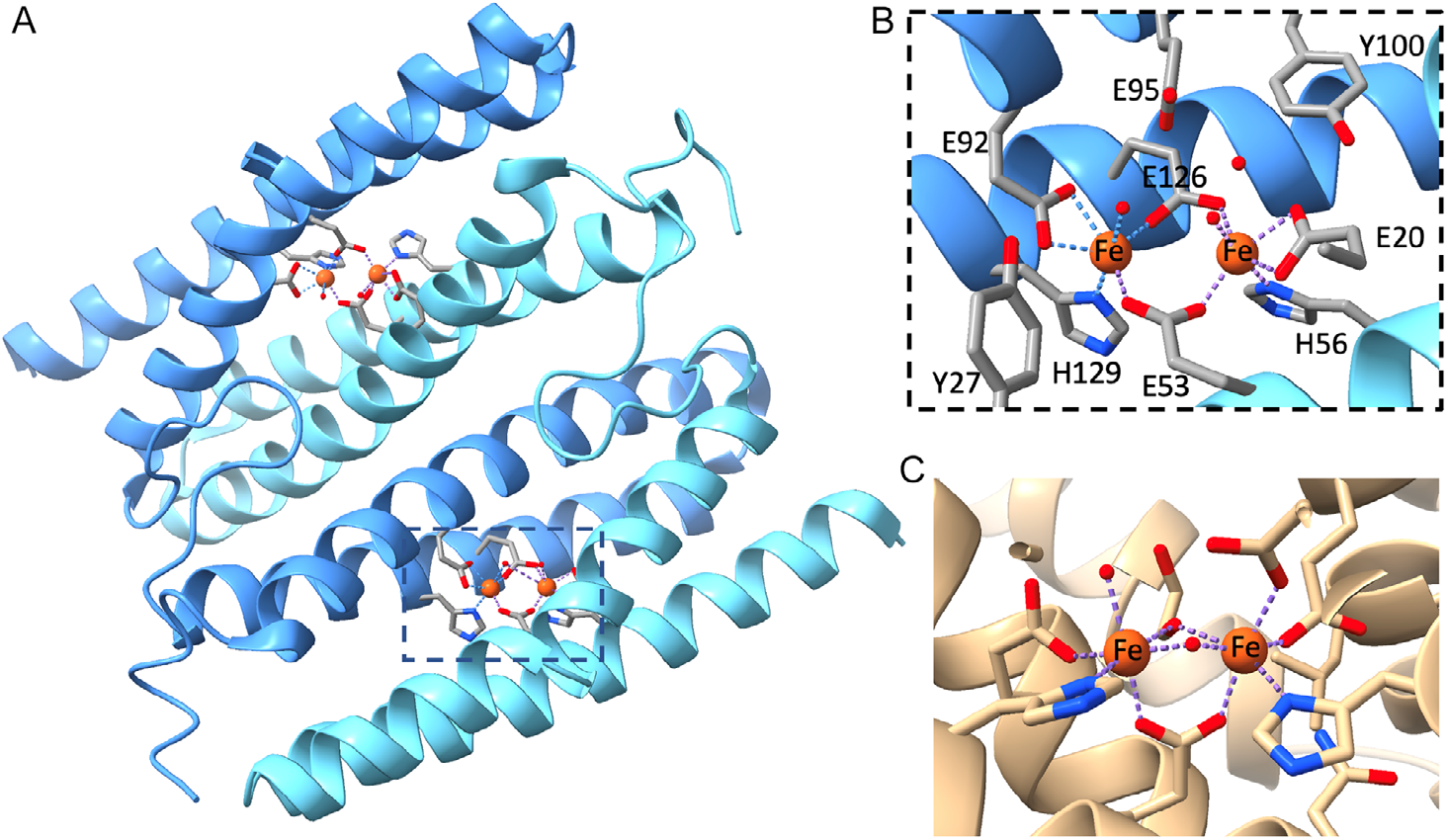
(A) Four‐helix bundle structure of diFe‐SulE from *Sulfolobus tokodaii* (PDB: 7O8D) showing the two diiron sites. (B) Close‐up of one Fe–Fe site with key amino acid side chains in gray licorice representation. (C) Diiron active site of *Methylosinus trichosporium* OB3b soluble methane monooxygenase hydroxylase (PDB: 6VK6).

Sulerythrins (SulE) are members of the ferritin‐like superfamily, found in obligate aerobic organisms such as *Sulfolobus tokodaii* [1, 2]. They adopt a domain‐swapped dimer structure with a four‐helix‐bundle (FHB) fold and a heterobimetallic Fe–Zn center (Figure 1) [1, 2, 3, 4]. SulE can be viewed as a truncated Rubrerythrin, lacking the C‐terminal rubredoxin domain containing a FeS_4_ motif [1, 2]. While SulE has been implicated in oxidative stress responses [2, 3, 5], its precise biological function remains unknown [1, 2, 3, 4, 5, 6]. The FHB scaffold of SulE accommodates various bimetallic sites—including Fe‐Zn, diFe, diNi, diMn, and diCo [6]—making it a versatile platform for studying metal incorporation in proteins and for designing novel metalloenzymes [6, 7, 8]. Among these, the diFe‐SulE variant is of particular interest due to its resemblance to widely studied diiron carboxylate proteins (Figure 1) [9, 10, 11].

The high‐resolution structure of reconstituted diFe‐SulE was first reported by Jeoung et al*.* [6]. The 1.12 Å structure shows a dimer, with each subunit contributing two *α*‐helices to form FHBs that harbor two bimetallic active sites (Figure 1A). These sites share a similar amino acid environment but differ slightly in Fe–Fe distances (3.70 Å for site 1, 3.76 Å for site 2) and metal occupancies. Spatially resolved anomalous dispersion (SpReAD) analysis revealed that one iron ion (Fe2) is more oxidized than the other, indicating a mixed‐valence state in the crystal [5]. Partial photoreduction during crystallography may account for minor differences from the original high‐resolution structure [6]. As in other diiron carboxylate proteins [9], including methane monooxygenase (MMO) [10, 11] and ribonucleotide reductase R2 (RNR R2) [12], the two Fe ions in diFe‐SulE are coordinated by two histidines and four glutamates. A fifth glutamate, located above the bimetallic center, forms short hydrogen bonds with the oxo ligands. Two bridging oxo groups are observed, similar to MMO [10] (Figure 1C), but their distances and occupancies differ between the two sites [5, 6]. This variability leads to ambiguity in defining the substrate and its conformation. Despite reconstitution in H_2_O_2_ the exact chemical nature of these oxo ligands remains uncertain [5].

Quantum‐chemistry‐based modeling has become essential in structural molecular biology [13]. Diiron non‐heme proteins have been widely studied using density functional theory (DFT) to optimize structures and probe the electronic properties of bimetallic active sites [13, 14, 15]. In particular, hybrid QM/MM approaches, combining quantum mechanics for the active site with molecular mechanics for the surrounding protein, provide an efficient and accurate way to capture both electronic and environmental effects [3, 16, 17, 18, 19, 20]. For open‐shell systems with strongly antiferromagnetically coupled spins, as often found in multinuclear metal cofactors, single‐determinant methods like DFT cannot fully describe the multideterminantal wavefunction [21]. The broken‐symmetry (BS) DFT approach, introduced by Noodleman in 1981 [22], approximates the multideterminantal state by averaging monodeterminantal wavefunctions of pure spin states with localized spin distributions [22, 23, 24]. This BS‐DFT approach has been successfully applied to many metalloenzymes over the past decades [3, 18, 24].

Therefore, the main goal of this work is to harness the power of hybrid BS‐DFT/MM calculations to complete and refine the 3D structure of the diFe‐SulE, elucidate the electronic properties of the diiron active site and determine the chemical nature of the dioxo species coordinating the iron ions. These calculations will advance our understanding of the chemical reactivity and catalytical properties of nonheme diiron proteins, thereby providing essential atomistic information for the rational design of new metalloenzymes.

## 2 Material and Methods

**FIGURE 2:**
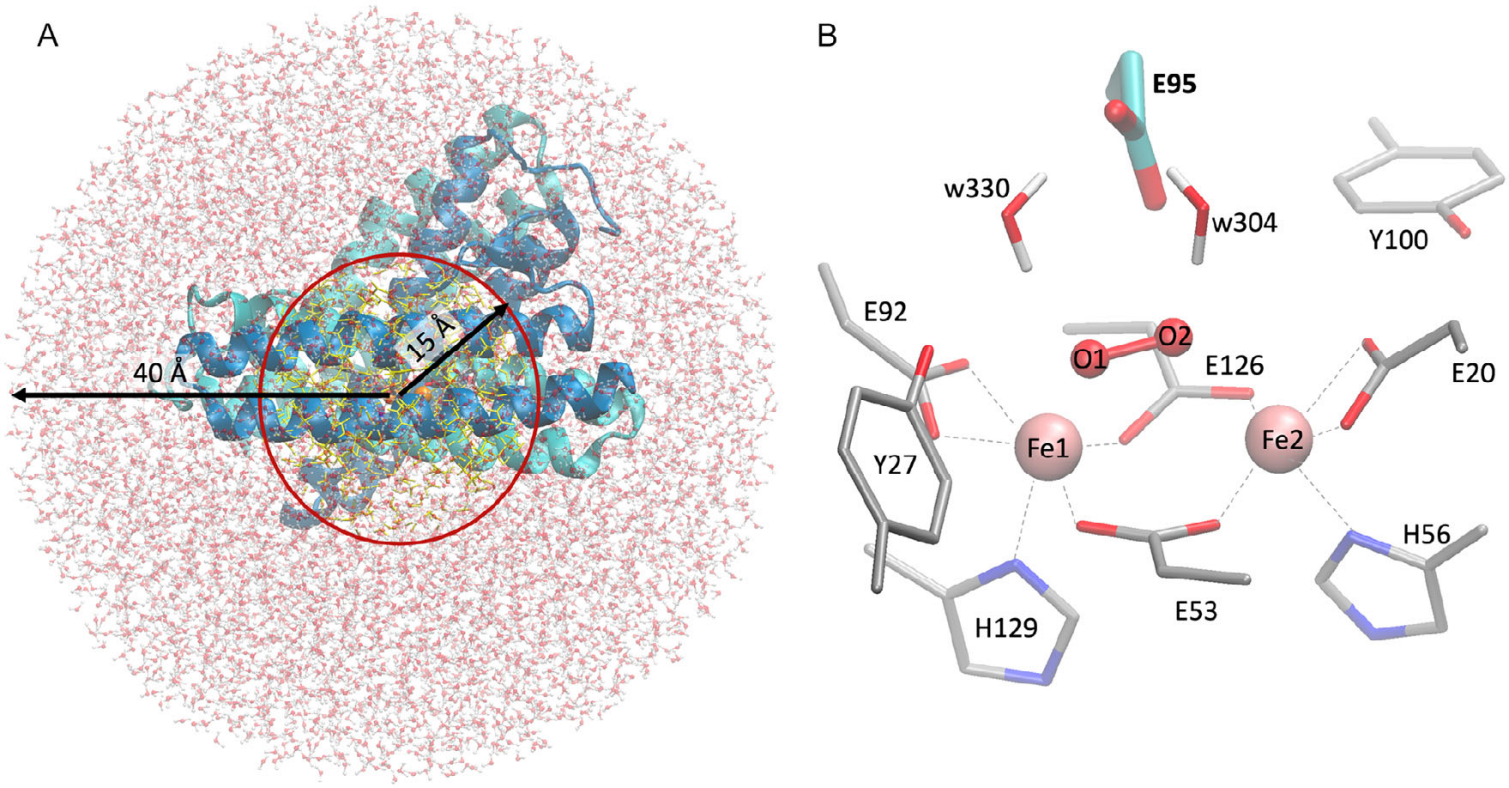
QM/MM setup. (A) diFe‐SulE embedded in a 40 Å water droplet centered on the chain A iron atom. The red circle indicates the 15 Å MM‐active region (yellow), outside of which atoms belong to the MM‐inactive region. (B) Close‐up view of the QM region, illustrating all heavy atoms of the active site together with nearby water molecules treated at the quantum‐mechanical level. Glu95, whose protonation state is a key focus of this study, is highlighted in cyan.

Structural models of diFe‐SulE were generated using a hybrid BS‐DFT/MM approach, in which the electronically relevant active site and its immediate environment are treated quantum mechanically, while the remainder of the protein and solvent are described using classical molecular mechanics [17]. The starting coordinates were taken from the high‐resolution dimeric diFe‐SulE crystal structure (PDB: 7O8D) [6]. Hydrogen atoms were added using the CHARMM hbuild tool [25] at pH 7, with His56 and His129 protonated at N*ε* due to their coordination to the iron ions. The protonation state of Glu95, which lies close to the diiron center and interacts with dioxygen species, was modeled in three variants: deprotonated (E95x), protonated at the distal oxygen Ot (E95t), or protonated at the proximal oxygen Op (E95p) (Figure 2) [3]. The dimeric protein, including the two diiron centers and crystallographic water molecules, was solvated in a TIP3P water [26] box with 0.1 M NaCl using VMD *solvate* plugin [27]. This workflow places a pre‐equilibrated water box around the protein. Short energy minimizations—consisting of an initial Steepest Descent run to remove steric clashes, followed by Adopted Basis Newton–Raphson (ABNR) minimization—were then performed under periodic boundary conditions to further relax the system and optimize the hydrogen‐bond network at the protein–solvent interface, using the CHARMM36 force field [28].

QM/MM calculations were carried out using the Chemshell software package [29]. The molecular system for BS‐DFT/MM calculations was defined as a 40 Å sphere centered on the Fe1 atom of site 1. The enzyme was partitioned into QM‐active, MM‐active, and MM‐inactive regions (Figure 2) [17]. Only atoms in the QM and MM‐active regions were optimized, while MM‐inactive atoms were fixed at crystallographic positions. The QM region included the diiron catalytic center that encompasses the Fe1 (residue number 201, chain B) and Fe2 (residue number 201, chain A) from site 1; the dioxo species (modeled as O_2_^2−^, H_2_O_2_, 2 H_2_O, or OOH^‐^); side chains of Glu20, Glu53, Glu92, Glu95, Glu126, His56, His129, Tyr27, and Tyr100; and two water molecules (w304 and w330) near Glu95, totaling 116–119 atoms depending on ligand and Glu95 protonation. BS‐DFT calculations were performed with Turbomole using B3LYP/def2‐TZVP for Fe and 6‐31G* for C, N, O, and H [30, 31], considering diFe(II), diFe(III), and mixed‐valence Fe(II)Fe(III) states. High‐spin states were used to generate broken‐symmetry Ms = 0 and Ms = 1/2 states, with total energies approximated from appropriate dimer spin combinations. QM/MM boundaries were treated with hydrogen link atoms and electrostatic embedding using a charge‐shift scheme [17]. Geometry optimizations employed hybrid delocalized internal coordinates with L‐BFGS quasi‐Newton algorithms [32]. A total of 36 structural models were generated, each in three spin states (hs, bs1, bs2), and the electronic and spin properties of the converged models are summarized in Table S1. Model names follow a systematic convention based on the oxidation state (diFe3, diFe2, Fe3Fe2), the ligand type (oho, o2, h2o2, 2h2o), and the protonation state of Glu95 (E95x, E95t, E95p). Each model was also assigned a unique index number for easy reference.

Protein geometries were rendered in VMD [27] and analyzed with custom Python scripts.

## 3 Results and Discussion

### 3.1 Overview of QM/MM‐Optimized Geometries of DiFe‐SulE

Most of the 36 initial models, optimized with the L‐BFGS algorithm, converged to local energy minima using criteria of 0.003 Bohr (maximum step) and 0.0008 Hartree/Bohr (maximum gradient). The structural models generally retained their initial configurations, with a few exceptions. In diFe2_oho_e95p, Fe3Fe2_oho_e95p, and diFe2_h2o2_e95p, the proximal hydrogen of Glu95 was abstracted by the dioxo ligand, generating the existing configurations diFe2_h2o2_e95x (4), Fe3Fe2_h2o2_e95x (22), and a new Fe3Fe2_oh_h2o_e95x model. In diFe3_oho_e95x, the hydroxide coordinated to Fe2 transferred a hydrogen to Glu95, converging to diFe3_o2_e95p (13). In other models, geometry optimization and wavefunction relaxation led to electron density redistribution between the metals and ligands, reflected in the updated model nomenclature. In total, 29 converged structural models (Table S1) were selected for further analysis.

**FIGURE 3:**
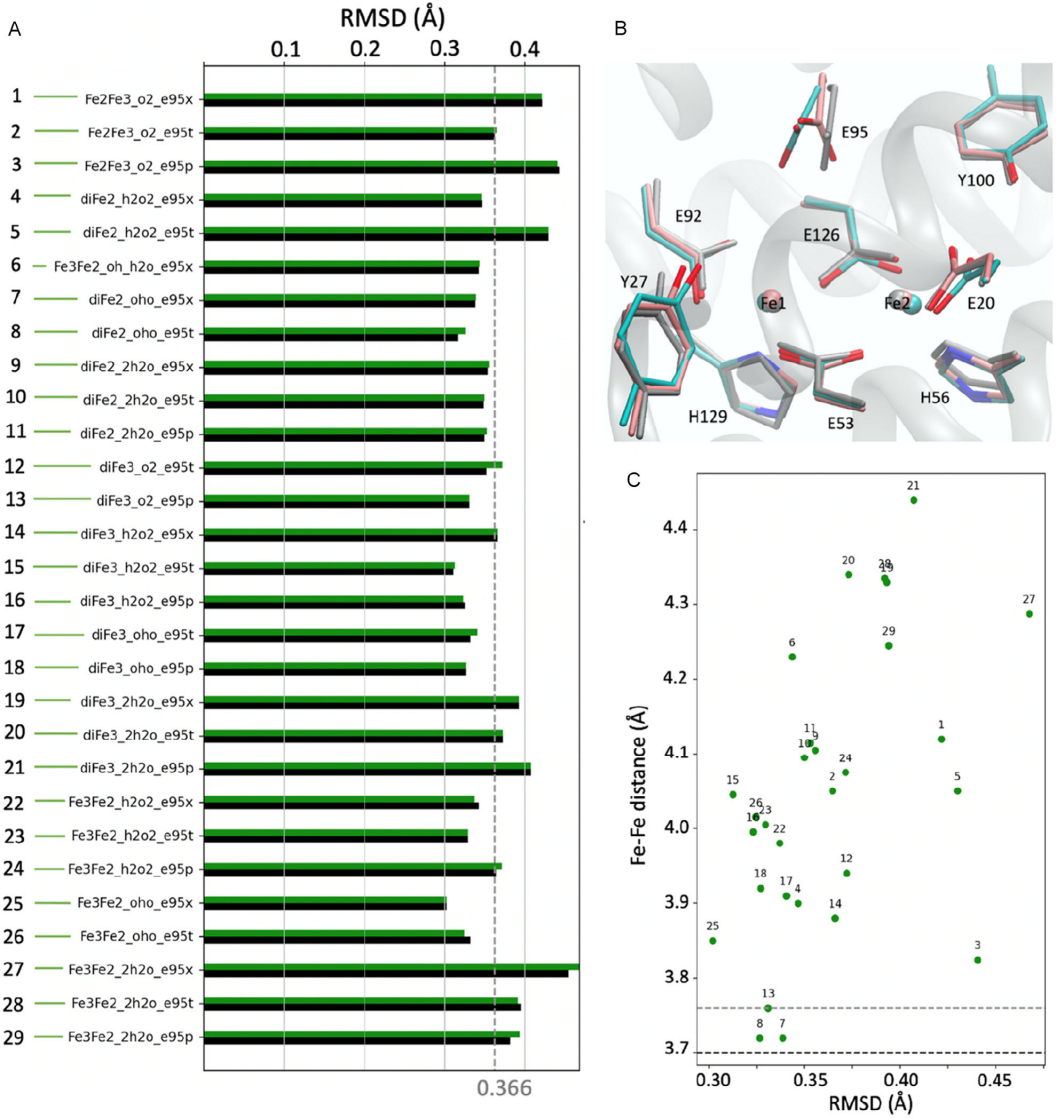
(A) RMSD of heavy atoms in 29 diFe‐SulE active site models in high‐spin (black) and broken‐symmetry (green) states relative to the crystal structure (PDB: 7O8D) [6]. (B) Overlay of high‐spin models Fe3Fe2_oho_e95x (pink, lowest RMSD) and Fe3Fe2_2h2o_e95x (cyan, highest RMSD) with the crystal structure (gray). Only atoms used for RMSD evaluation are shown. (C) Predicted Fe–Fe distances (Å) and RMSD values for all 29 models. Dashed lines mark experimental Fe–Fe distances in site1 (black) and site 2 (gray).

Despite variations in model configurations, the optimized structures differ only slightly from the crystal structure, with RMSD values below 0.50 Å (Figure 3). RMSDs were computed over all heavy atoms of the active site cavity‐excluding the dioxygen species ‐ using the crystal geometry (PDB: 7O8D) as reference. The cavity includes the diiron center and side chains of Glu95, Glu92, Glu20, Glu126, Glu53, His129, His56, Tyr27, and Tyr100. The lowest RMSD is 0.302 Å for Fe3Fe2_oho_e95x (25), while the highest (∼0.50 Å) occurs for Fe3Fe2_2h2o_e95x (27) models. These differences mainly arise from subtle rotations of Glu95 and Tyr100 due to hydrogen‐bonding variations (Figure 3B) and remain within the crystallographic resolution (1.12 Å) [6]. Therefore, selecting a “best” model based solely on RMSD is not possible. However, a trend is observed: Models with an O_2_^2−^ ligand or two water molecules induce larger distortions (RMSD > 0.40 Å) by rotating Glu95, whereas models with a μOH–O ligand (RMSD ∼0.30 Å) or hydrogen peroxide—with Glu95 protonated at the t‐position—show better agreement with the crystal structure.

Fe–Fe distances in the active site were measured to assess structural properties (Figures 3C and S1). QM/MM optimizations show that these distances are influenced by the metal redox state [33], the protein environment [34], and the nature of the ligating species. Depending on the model, Fe–Fe distances range from 3.71 Å (diFe2_oho_e95x (7)) to 4.44 Å (diFe3_2h2o_e95p (21)). In H_2_O_2_‐treated diFe‐SulE, XRD reports distances of 3.70 Å (site 1) and 3.76 Å (site 2) [6], while EXAFS shows a broader range from 3.37 Å (solution) to 4.02 Å (as‐isolated crystal), reflecting experimental conditions and possible partial photoreduction during data collection [35, 36, 37].

The Fe1–Fe2 distance at site 1 is best reproduced by models featuring a di‐μ‐hydroperoxo bridge between ferrous centers (diFe2_oho_e95x (7) and diFe2_oho_e95t (8)), which predict ∼3.72 Å and exhibit low RMSD values. In these models, Glu95 is either negatively charged or protonated, indicating that the side chain charge alone does not determine the Fe–Fe separation. For site 2, the Fe1–Fe2 distance of ∼3.76 Å is accurately captured by the diFe(III) model coordinating O_2_^2‐^ (diFe3_o2_e95p (13)), where the oxygen is stabilized through weak hydrogen bonds with protonated Glu95 and a nearby water molecule. Notably, none of the QM/MM models converge to distances below 3.5 Å, as suggested by EXAFS for H_2_O_2_‐treated diFe‐SulE [6]. This discrepancy likely arises from the end‐on μ‐oxo bridge in diFe‐SulE, in contrast to the side‐to‐side “diamond” arrangement observed for the Fe_2_O_2_core in soluble methane monooxygenase (sMMO) and ribonucleotide reductase RNR‐R2 [33, 38, 39]. Overall, the Fe–Fe distances in diFe‐SulE are larger than in sMMO (3.10–3.40 Å) [33] but comparable to RNR‐R2. In RNR‐R2 ligand bridging leads to a Fe–Fe distance shortening from 3.90 Å to 3.40 Å in the structure of the reduced wild‐type protein [39] and involves a significant rearrangement of one of the bridging carboxylates (a manifestation of the “carboxylate shift” [40], which is one important source of the coordinative flexibility) in addition to the bridging and terminal solvent ligands [9].

The ∼0.40 Å Fe–Fe shortening upon one‐electron oxidation proposed by Jeoung et al. [6] is partially supported by our QM/MM results. The Fe–Fe distance difference between the mixed‐valence Fe2Fe3_h2o2_e95x (22) and the diferric diFe3_h2o2_e95x (14) or diFe3_oho_e95t (17) models is ∼0.10 Å, exceeding the mean B3LYP deviation (MD = 0.049 Å) [41, 42] and indicating a genuine structural effect. However, this trend is not universal, as the diiron geometry also depends on subtle environmental variations (Figure S1). For instance, oxidation of diFe2_oho_e95x (7) to Fe3Fe2_oho_e95x (25) or diFe2_oho_e95t (8) to Fe3Fe2_oho_e95t (26) leads to Fe–Fe elongation of up to 0.3. Å (Figure S3).

Overall, RMSD and Fe–Fe analyses suggest that the crystal structure of diFe‐SulE represents two states: a fully reduced diFe(II) center with a di‐μ‐hydroperoxo (μOH–O) bridge at site 1 and a diferric center coordinated to O_2_^2−^ at site 2. Protonation of Glu95 further modulates the hydrogen‐bonding network and subtly alters Fe–Fe interactions (Figure S1).

### 3.2 Chemical Nature of the Bridging Ligand

**FIGURE 4:**
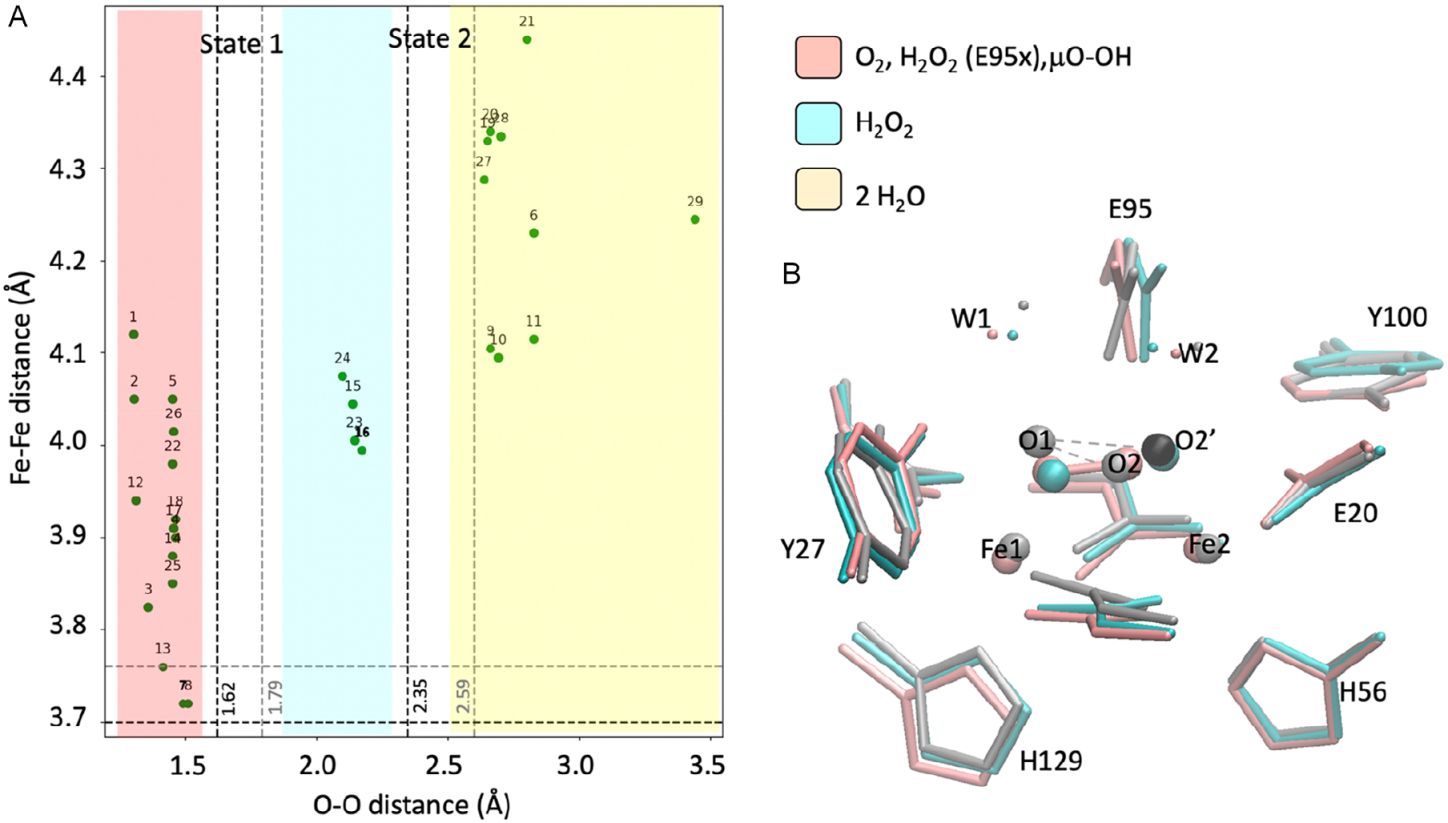
(A) Correlation between O–O and Fe–Fe distances from QM/MM‐optimized diFe‐SulE models. Dashed lines mark experimental values for sites 1 (black) and 2 (gray) [6]; shaded regions indicate ligand types. (B) Overlay of models diFe2_oho_e95p (pink) and diFe3_h2o2_e95p (cyan) with the crystal structure (PDB: 7O8D, gray) [6]. Gray and black spheres show O2 positions in states 1 and 2, respectively. Model 7 represents state 1, and model 16 represents state 2. Distances in Å.

The crystal structure of Fe^2+^‐reconstituted SulE [6] reveals a dioxo ligand bridging the two iron ions in an *end‐on* fashion, though its precise identity remains unclear. By analogy with rubrerythrin‐like proteins [43], this ligand was tentatively modeled as hydroxide ions. The experimental O–O distances of 1.62 Å (site 1) and 1.79 Å (site 2) in diFe‐SulE fall within the 1.47–1.80 Å range reported for H_2_O_2_‐treated *P. furiosus* rubrerythrin (PDB: 3PWF, 3PZA, 3QVD) [43], but exceed typical O–O bond lengths in isolated H_2_O_2_ (1.48 Å) [44] or peroxo‐diferric complexes (1.39–1.43 Å) [45, 46], suggesting an *end‐on* di‐μ‐hydroperoxo bridge. Moreover, the H_2_O_2_‐treated diFe‐SulE structure shows two O2 positions—one at 1.7 Å (33% occupancy) and another at 2.5 Å (67% occupancy) away from O1—indicating two coexisting states differing in the nature of the dioxo species. We therefore designate *state 1* as the short O–O configuration and *state 2* as the long O–O configuration (Figure 4).

Analysis of the O1–O2 distances in the 29 QM/MM‐optimized models revealed three structural groups (Figure 4). Group 1 includes models with *end‐on* di‐μ‐peroxo, di‐μ‐hydroperoxo, or μ‐hydroperoxide ligands and a deprotonated Glu95, characterized by short O–O bonds (<1.50 Å). Group 2 comprises μ‐H_2_O_2_ models with protonated Glu95 and intermediate O–O distances around 2.30 Å, while Group 3 contains H_2_O‐coordinated models showing long O–O separations (>2.50 Å). A clear correlation was observed between O–O and Fe–Fe distances: models in Group 1 span 3.70– 4.40 Å, those in Group 2 cluster around 4.00–4.10 Å, and Group 3 exceeds 4.1 Å. Relative to the experimental values (indicated in Figure 4 by dashed lines for both active sites and oxidation states) [6], the DFT‐optimized structures systematically underestimate the O–O bond lengths by approximately 0.1–0.3 Å. Overall, the results support assigning state 1 to an *end‐on* di‐μ‐hydroperoxo or di‐μ‐peroxo bridge at sites 1 and 2, respectively, consistent with low RMSD values and experimental Fe–Fe distances (∼3.70 Å). State 2, with longer O–O separations (>2.30 Å), likely corresponds to a diferric center bridged by a μ‐hydroperoxo ligand and a protonated Glu95. Finally, the presence of water in the diiron active site appears to be unlikely. Water molecules directly interacting with the iron ions not only push the metals apart, resulting in very long Fe1‐Fe2 distances exceeding 4.10 Å, but also cause reorientation of side chains at the active site. This is evidenced by RMSD values for heavy atoms exceeding 0.4 Å.

### 3.3 Exchange Coupling Between Iron Sites

The magnetic interaction between the two iron centers with magnetic spins *S*_Fe1_ and *S*_Fe2_ was estimated using the exchange coupling constant *J*, defined by the Heisenberg–Dirac–van Vleck Hamiltonian [47, 48, 49]:

(1) $$H=-2J\left(S_{\mathrm{Fe}1}\cdot S_{\mathrm{Fe}2}\right)$$

When the high‐spin (*E*_HS_) and broken‐symmetry (*E*_BS_) energies are known, *J* can be calculated as [50]:

(2) $$J=\frac{2}{n_{\mathrm{Fe}1}n_{\mathrm{Fe}2}}\left[E_{\mathrm{BS}}-E_{\mathrm{HS}}\right]$$

where *n*_Fe1_ and *n*_Fe2_ are the numbers of unpaired electron in each iron center. Thus, using QM/MM‐optimized geometries and corresponding energies for both spin states, we estimated *J* between the two iron ions (Table S1 and Figure S2). Most models show low coupling constants (<100 cm^−1^), spanning −471 to 214 cm^−1^ depending on the bridging ligand and local environment, reflecting their sensitivity to structural variations. Models with Fe–Fe distances <4.0 Å exhibit weak antiferromagnetic or nearly diamagnetic behavior (*J* > 15 cm^−1^), consistent with other oxo‐bridged diiron complexes [42]. Strong antiferromagnetic coupling appears only in a few models—diFe2_oho_e95t (8), Fe2Fe3_o2_e95p (3), and diFe3_o2_e95t (12)—all featuring protonated Glu95. Remarkably, protonation of Glu95 (Ot) in diFe2_oho_e95t (8) increases *J* to −470 cm^−1^ compared with negligible coupling in the unprotonated diFe2_oho_e95x (7). As spin densities are similar in both, this large *J* likely reflects most likely a QM/MM artifact from the complex electronic–environmental interplay. Experimental confirmation is still lacking.

## 4 Discussions

**FIGURE 5:**
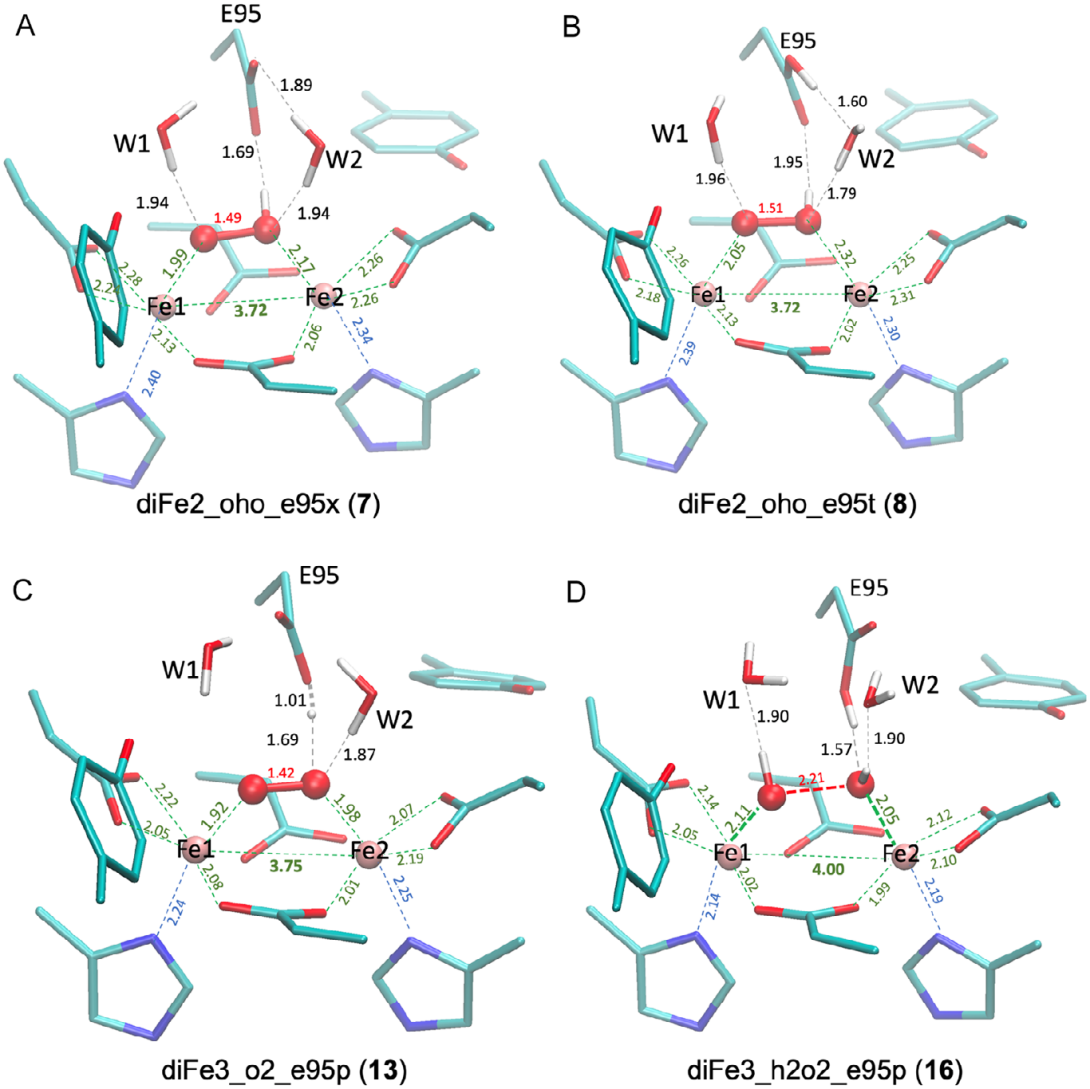
QM/MM‐optimized diFe‐SulE active sites. (A,B) Active site 1 and (C) site 2 in state 1 with a short O–O distance of the dioxo ligand. (D) Active site 1 in state 2 showing an elongated O–O bond indicative of activation.

Starting from the crystal structure of H_2_O_2_‐treated diFe‐SulE, we conducted a thorough analysis of the structural and electronic properties of 29 different models. These models varied in the protonation state of Glu95, the chemical nature of the bridging dioxo species, and the oxidation state of the metal ions. This analysis yielded four potential QM/MM models that best match the experimental crystal structure of the active sites of diFe Sul E (Figure 5, Table 1). Although diFe SulE forms a homodimer, with each monomer containing a binuclear metal center, the crystal structures reveal slight differences in the atomic arrangement [5, 6]. This suggests that the two active sites may exist in different states [6]. Additionally, the dioxo ligand can be found in two distinct conformations, each characterized by different O–O distances, indicating the presence of different bridging ligands. Consequently, the crystal structure of the active site in H_2_O_2_‐treated diFe SulE is heterogeneous, with at least three different states potentially coexisting. In the following, we will provide a detailed description of the structural models resulting from the QM/MM calculations that best characterize each of these states.

**TABLE 1:** Structural and electronic parameters of QM/MM diFe‐SulE models for active sites 1 and 2 in states 1 (short O–O) and 2 (long O–O), including model name, RMSD (Å), Fe–Fe and O–O distances (Å), and energies of high‐spin (Ms = 8, Ms = 10) and broken‐symmetry (Ms = 0) states (Hartree) with their differences.

Active site	Models	RMSD	Fe–Fe	O–O	*E*_BS_	*E*_HS_	Δ*E*
Site 1, State 1	diFe2‐oho‐e95t (8) diFe2‐oho‐e95x (7)	0.316 0.338	3.72 3.72	1.51 1.49	−5525.5896 −5525.6263	−5525.5724 −5525.6264	−0.0172 0.0001
Site 2, State 1	diFe3_o2_e95p (13)	0.331	3.75	1.42	−5523.7266	−5523.7249	−0.0016
Site 1, State 2	diFe3_h2o2_e95p (16)	0.326	4.00	2.21	−5524.4996	−5524.4973	0.0005

*Active site 1:* The active site 1 of the diFe‐SulE is characterized by a Fe–Fe distance of only 3.70 Å and a O–O distance of 1.62 Å. According to QM/MM geometry optimizations, this site would be best described by a diferrous metal center, in which each iron is coordinated by four glutamate residues and a histidine residue. A μ‐hydroperoxo ligand mainly coordinated to Fe1 bridges both iron centers. ‐One‐electron oxidation of Fe1, as predicted in models Fe3Fe2_oho_e95x (25) and Fe3Fe2_oho_e95t (26), leads to a 0.1–0.3 Å elongation of the Fe–Fe distance, driven by increased electrostatic repulsion between the metal centers. As shown in Figure S3, this subtle change weakens the interaction between ferrous Fe2 and the μ‐hydroperoxo ligand, increasing the Fe2–O2 distance by approximately 0.4 Å, and induces minor conformational adjustments in the surrounding environment. The μ‐hydroperoxo bridge is further stabilized by hydrogen bond interaction with the carbonyl oxygen of the Glu95 (Figure 5A,B). The orientation of water molecule W2 dictates the protonation state of Glu95, allowing it to exist either in its deprotonated (anionic, E95X) or protonated (E95T) form. This seemingly small change has a substantial structural and energetic impact: altering the protonation state reorganizes the local hydrogen‐bonding network and strongly affects the stability of the system.

Our QM/MM calculations show that the anionic form of Glu95 is favored by 32 kcal/mol relative to the protonated form. This value was obtained from the energy difference between models Fe3Fe2_oho_e95x (25) and Fe3Fe2_oho_e95t (26), corrected by the standard free energy of protonation (3.00 kcal/mol), assuming a pKa of 4.88 for the propionic side chain at T = 298 K and pH 7 [50]. The Fe–Fe separation is only marginally affected by the surrounding environment, and the spin‐density distribution on both metal centers remains essentially unchanged. Population analysis yields spin densities of 3.76 and 3.77 e‐ on Fe1 and Fe2 (high spin state), respectively (Table S2). We therefore conclude that the strong antiferromagnetic (J of −470 cm^−1^) predicted for diFe2‐oho‐e95t (8) most likely arises from a delicate interplay between the electronic structure and the protein environment. This interpretation is supported by the negligible geometric differences between the high‐spin and broken‐symmetry solutions (RMSD of coordinating heavy atoms ≈0.02 Å). Analyzing the charge and spin distribution in both models (diFe2‐oho‐e95t (8) and diFe2‐oho‐e95x (7)) reveals a nearly balanced charge on the Fe atoms (∼0.75 a.u.) suggesting that both Fe atoms have a similar affinity for binding to the dioxygen species.

The second conformation of the bridging oxygen (state 2) characterized by a O–O distance of 2.50 Å can be best described by models harboring hydrogen peroxide ligand. However, all structures with H_2_O_2_ show slightly longer Fe–Fe distances (4.00 < d_Fe–Fe_ < 4.10 Å) compared with the values of 3.70 and 3.89 Å reported for the crystallographic structure of SulE reconstituted with Fe^2+^ and the corresponding EXAFS fitted parameters [6]. The lowest RMSD values relative to the active site of the crystal structure is predicted for model diFe3_h2o2_e95t (16) alluding to a diferric state of the iron centers each coordinating a hydroxo ligand interacting with a protonated Glu95 (Figure 5D). The presence of water molecules breaks the symmetry of the active site as reflected by the slightly different interatomic distances and charge densities at the iron centers (Table S2). This model for state 2 (diFe3_h2o2_e95t (16)) most likely represents an intermediate state in the Fenton mechanism for H_2_O_2_ disproportionation in which O–O bond cleavage has occurred and the resulting hydroxyl radical on Fe2 and hydroxide anion on Fe1 are stabilized by the two ferric centers and the surrounding hydrogen bonding network [51].

*Active site 2:* The active site 2 of the diFe SulE, on the other hand, is characterized by a Fe–Fe distance of 3.77 Å and a O–O distance of 1.7 Å. According to QM/MM geometry optimizations, this arrangement is best reproduced by model diFe3_o2_e95p (13) with a diferrous iron center and a protonated Glu95 (Figure 5C). The dioxo unit is best described as a di‐μ‐peroxo bridge**,** in which one of the O^‐^ ligands shares a proton with the carboxylic side chain of Glu95. The O–O bond length is 1.42 Å, consistent with values observed in peroxo‐diferric complexes (1.39–1.43 Å) [45, 46]. Although the coordination environments of the two iron centers are largely similar, the hydrogen bond involving Glu95 induces subtle conformational adjustments that generate a slight electronic asymmetry, favoring oxidation at Fe2 over Fe1. Analysis of the charge distribution in diFe3_o2_e95p (13) reveals a marginally higher charge density on Fe2 (0.99 a.u.) compared to Fe1 (0.97 a.u.) (Table S2). The spin densities, however, remain nearly identical (Δ*ρ*(Fe1) = 4.22 and Δ*ρ*(Fe2) = 4.21), supporting a diferric rather than a mixed‐valence description, in contrast to the interpretation derived from the SpReAD analysis by Lennartz et al*.* [5]. This modest electronic polarization may nevertheless influence substrate activation, highlighting the sensitivity of the diiron site to its hydrogen‐bonding environment.

## 5 Conclusion

Our QM/MM investigation of 29 structural models derived from the crystal structure of H_2_O_2_‐treated diFe‐SulE reveals that the enzyme's diiron active site is structurally and electronically heterogeneous, accommodating at least three distinct states. The best agreement with experimental data was obtained for four optimized models that reproduce the Fe–Fe and O–O distances observed crystallographically. Active site 1 is best represented by a diferrous center bridged by an *end‐on* di‐μ‐hydroperoxo ligand, with Glu95 either deprotonated or protonated depending on the local hydrogen‐bonding network. Variations in Glu95 protonation subtly influence the Fe–Fe distance and electronic coupling, but the overall coordination geometry and spin distribution remain largely conserved. Upon one‐electron oxidation, the Fe–Fe distance increases slightly, consistent with enhanced electrostatic repulsion between the iron centers. Active site 2, in contrast, is best modeled as a diferric center bridged by a di‐μ‐peroxo ligand stabilized through hydrogen bonding to protonated Glu95. This configuration reproduces the experimentally observed Fe–Fe distance and the characteristic O–O bond length of peroxo‐bridged diiron complexes. The small electronic asymmetry between the iron centers suggests a potential role of hydrogen‐bonding interactions in modulating redox reactivity and substrate activation. Overall, the QM/MM results indicate that the crystallographic structure of diFe‐SulE captures an ensemble of coexisting redox and protonation states, reflecting the intrinsic flexibility of its diiron center. This heterogeneity likely underlies the enzyme's functional adaptability in oxidative catalysis and supports a mechanistic model involving dynamic interconversion between diferrous, diferric, and mixed‐valence species during peroxide activation.

## Supporting Information

Supplementary data (Table S1 and S2 and Figure S1, S2, and **S3**) are given in the Supporting Information. Additional supporting information can be found online in the Supporting Information section. **Supporting Fig. S1:** Fe‐Fe distances (Å) from QM/MM optimized structural models of diFe SulE in high‐spin (black bars) and broken‐symmetry (green bars) states. The black and gray broken lines indicate the Fe‐Fe distances of 3.70 Å and 3.76 Å measured in the crystal samples, for site 1 and site 2, respectively [6]. **Supporting Fig. S2: Magnetic exchange coupling (J, cm** ^**−1**^ **)** as a function of the **Fe–Fe distance** (Å) for QM/MM‐optimized diiron models with Fe–Fe separations below 4.0 Å. The yellow‐shaded area marks the regime of very weak coupling (|J|<15 cm^−1^), corresponding to an effectively diamagnetic diiron site. **Supporting Fig. S3:** Comparison of high‐spin structural models of active site 1 of diferrous SulE (left panels) with the corresponding mixed‐valence Fe(III)–Fe(II) models (right panels), shown for both the deprotonated (anionic) Glu95 (upper panels) and protonated Glu95 (lower panels). The RMSD between the diferrous and mixed‐valence models, calculated over all heavy atoms in the QM region, is 0.20 Å for both protonation states of Glu95. **Supporting Table S1:** List of converged QM/MM models of the catalytic site of diFe‐SulE and their key electronic properties. Model names in brackets indicate the original electronic configurations. Optimized QM/MM models that best reproduce the experimental structure are shown in **bold**. **Supporting Table S2:** Charge (q) and spin density (Δ*ρ*) at Fe1 and Fe2 sites of the QM/MM optimized models of diFeSulE derived from population analysis with Turbomole.

## Author Contributions

**Samah Moubarak**: formal analysis (equal), investigation (lead), and methodology (lead). **Maria Andrea Mroginski**: conceptualization (lead), formal analysis (equal), funding acquisition (lead), investigation (equal), methodology (supporting), project administration (lead), resources (lead), validation (lead), visualization (equal), writing original draft (lead), and writing – review and editing (lead).

## Conflicts of Interest

The authors declare no conflicts of interest.

## Supporting information

Supplementary Material

## Data Availability

The data that support the findings of this study are available in the supplementary material of this article.
