# Supplementary material for: Electronic and Structural Heterogeneity in the Diiron Center of Sulerythrin: Insights From Hybrid QM/MM Calculations
Source: Chemphyschem. 2026 Jan 25;27(2):e202500772. doi: 10.1002/cphc.202500772 (PMC12833474; doi:10.1002/cphc.202500772)

Supplementary Information

Electronic and Structural Heterogeneity in the Diiron Center of Sulerythrin: Insights from Hybrid QM/MM Calculations

Samah Moubarak, Maria Andrea Mroginski*

Institut für Chemie- Technische Universität Berlin

E-mail: andrea.mroginski@tu-berlin.de

Table S1. List of converged QM/MM models of the catalytic site of diFe-SulE and their key electronic properties. Model names in brackets indicate the original electronic configurations. Optimized QM/MM models that best reproduce the experimental structure are shown in **bold**.

	Model	ligand	E95	# atoms	Total charge	Spin	Energy (a.u.)	J (cm^{-1})
1	Fe2Fe3_o2_e95x	O_2	dep.	116	-1	4 (hs)	-5521.507157	-3
	(diFe2_o2_e95x)					0 (bs)	-5521.507284	
2	Fe2Fe3_o2_e95t	O_2	prot.	117	0	4 (hs)	-5523.581884	-2
	(diFe2_o2_e95t)		O_t			0 (bs)	-5523.581954	
3	Fe2Fe3_o2_e95p	O_2	prot.	117	0	4 (hs)	-5522.814841	-122
	(diFe2_o2_e95p)		O_p			0 (bs)	-5522.819290	
4	diFe2_h2o2_e95x	H_2O_2	dep.	118	-1	4 (hs)	-5524.676325	-9
						0 (bs)	-5524.676663	
5	diFe2_h2o2_e95t	H_2O_2	prot.	119	0	4 (hs)	-5523.945220	-2
			O_t			0 (bs)	-5523.945287	
6	Fe3Fe2_oh_h2o_e95x	H_2O+OH	dep.	119	0	4 (hs)	-5524.234452	-22
	(diFe2_h2o2_e95p)					0 (bs)	-5524.235289	
**7**	**diFe2_oho_e95x**	OH+O^-	dep.	117	-2	4 (hs)	-5525.626362	4
						0 (bs)	-5525.626225	
**8**	**diFe2_oho_e95t**	OH+O^-	prot.	118	-1	4 (hs)	-5525.572447	-470
			O_t			0 (bs)	-5525.589594	
9	diFe2_2h2o_e95x	2 H_2O	dep.	120	-1	4 (hs)	-5525.674729	-14
						0 (bs)	-5525.675251	

10	diFe2_2h2o_e95t	2 H_2O	prot.	121	0	4 (hs)	-5525.859602	-221
			O_t			0 (bs)	-5525.867675	
11	diFe2_2h2o_e95p	2 H_2O	prot.	121	0	4 (hs)	-5526.961873	-67
			O_p			0 (bs)	-5526.964309	
12	diFe3_o2_e95t	O_2	prot.	117	2	5 (hs)	-5522.7217946	-199
			O_t			0 (bs)	-5522.7331285	
13	**diFe3_o2_e95p**	O_2	prot.	117	2	5 (hs)	-5523.724914	-29
			O_p			0 (bs)	-5523.726563	
14	diFe3_h2o2_e95x	H_2O_2	dep.	118	1	5 (hs)	-5523.0190519	-12
						0 (bs)	-5523.019744	
15	diFe3_h2o2_e95t	H_2O_2	prot.	119	2	5 (hs)	-5524.8838700	9
			O_t			0 (bs)	-5524.883349	
16	diFe3_ h2o2_e95p	H_2O_2	prot.	119	2	5 (hs)	-5524.497268	-40
			O_p			0 (bs)	-5524.499555	
17	diFe3_oho_e95t	OH + O^-	prot.	118	1	5 (hs)	-5525.8157818	-3
			O_t			0 (bs)	-5525.815997	
18	diFe3_oho_e95p	OH + O^-	prot.	118	1	5 (hs)	-5525.383233	-4
			O_p			0 (bs)	-5525.383466	
19	diFe3_2h2o_e95x	2 H_2O	dep.	120	1	5 (hs)	-5525.074416	-2
						0 (bs)	-5525.074543	
20	diFe3_2h2o_e95t	2 H_2O	prot.	121	2	5 (hs)	-5525.1070887	-9
			O_t			0 (bs)	-5525.1075770	
21	diFe3_2h2o_e95p	2 H_2O	prot.	121	2	5 (hs)	-5526.0207112	-5
			O_p			0 (bs)	-5526.0210060	
22	Fe2Fe3_h2o2_e95x	H_2O_2	dep.	118	0	9/2 (hs)	-5525.9156049	1
						½ (bs)	-5525.915567	
23	diFe3_ h2o2_e95t	H_2O_2	prot.	119	1	9/2 (hs)	-5524.543759	-5
	(Fe2Fe3_ h2o2_e95t)		O_t			½ (bs)	-5524.543990	
24	diFe3_ h2o2_e95p	H_2O_2	prot.	119	1	9/2 (hs)	-5524.287902	-199
	(Fe2Fe3_ h2o2_e95p)		O_p			½ (bs)	-5524.296907	

25	Fe3Fe2_oho_e95x	OH + O^-	dep.	117	-1	9/2 (hs)	-5525.479046	-6
	(Fe2Fe3_oho_e95x)					½ (bs)	-5525.479338	
26	Fe3Fe2_ oho_e95t	OH + O^-	prot.	118	0	9/2 (hs)	-5523.408555	-182
	(Fe2Fe3_ oho_e95t)		O_t			½ (bs)	-5523.416900	
27	Fe2Fe3_2h2o_e95x	2 H_2O	dep.	120	0	9/2 (hs)	-5526.117311	213
						½ (bs)	-5526.107571	
28	Fe2Fe3_2h2o_e95t	2 H_2O	prot.	121	1	9/2 (hs)	-5525.306288	-61
						½ (bs)	-5525.309077	
			O_t					
29	Fe2Fe3_2h2o_e95p	H_2O_2	prot.	121	1	9/2 (hs)	-5524.805039	-124
			O_p			½ (bs)	-5524.810689	

Table S2: Charge (q) and spin density (Δρ) at Fe1 and Fe2 sites of the QM/MM optimized models of diFeSulE derived from population analysis with Tubomole.

	Model name	q(Fe1)	q(Fe2)	Δρ(Fe1)	Δρ(Fe2)
1	Fe2Fe3_o2_e95x	0.72	0.96	3.77	4.12
2	Fe2Fe3_o2_e95t	0.74	1.03	3.78	4.21
3	Fe2Fe3_o2_e95p	0.73	0.99	3.77	4.20
4	diFe2_h2o2_e95x	0.77	0.74	3.78	3.78
5	diFe2_h2o2_e95t	0.75	0.76	3.78	3.78
6	Fe3Fe2_oh_h2o_e95x	1.03	0.83	4.26	2.86
**7**	**diFe2_oho_e95x**	**0.75**	**0.75**	**3.75**	**3.77**
**8**	**diFe2_oho_e95t**	**0.76**	**0.75**	**3.76**	**3.77**
9	diFe2_2h2o_e95x	0.74	0.76	3.78	3.78
10	diFe2_2h2o_e95t	0.78	0.72	3.78	3.78
11	diFe2_2h2o_e95p	0.78	0.73	3.79	3.78
12	diFe3_o2_e95t	0.97	0.99	4.20	4.19
**13**	**diFe3_o2_e95p**	**0.97**	**0.98**	**4.22**	**4.21**
14	diFe3_h2o2_e95x	0.99	1.05	4.26	4.24
15	diFe3_h2o2_e95t	0.99	0.99	4.24	4.24
**16**	**diFe3_h2o2_e95p**	**0.99**	**1.00**	**4.26**	**4.23**
17	diFe3_oho_e95t	0.97	0.99	4.20	4.23
18	diFe3_oho_e95p	0.99	0.99	4.22	4.23
19	diFe3_2h2o_e95x	0.99	1.05	4.26	4.24
20	diFe3_2h2o_e95t	1.00	1.04	4.26	4.23
21	diFe3_2h2o_e95p	1.02	1.04	4.20	4.25
22	Fe2Fe3_h2o2_e95x	0.77	1.04	3.80	4.23
23	diFe3_h2o2_e95t	0.97	1.02	4.23	4.25
24	diFe3_h2o2_e95p	1.01	1.00	4.25	4.23
25	Fe3Fe2_oho_e95x	1.01	0.76	4.16	3.78
26	Fe3Fe2_oho_e95t	1.04	0.72	4.20	3.78

27	Fe2Fe3_2h2o_e95x	0.77	1.04	3.80	4.23
28	Fe2Fe3_2h2o_e95t	0.76	1.03	3.79	4.22
29	Fe2Fe3_2h2o_e95p	0.77	1.02	3.79	4.23

Figure S1: Fe-Fe distances (Å) from QM/MM optimized structural models of diFe-SulE in high-spin (black bars) and broken-symmetry (green bars) states. The black and grey broken lines indicate the Fe-Fe distances of 3.70 Å and 3.76 Å measured in the crystal samples, for site 1 and site 2, respectively [6].

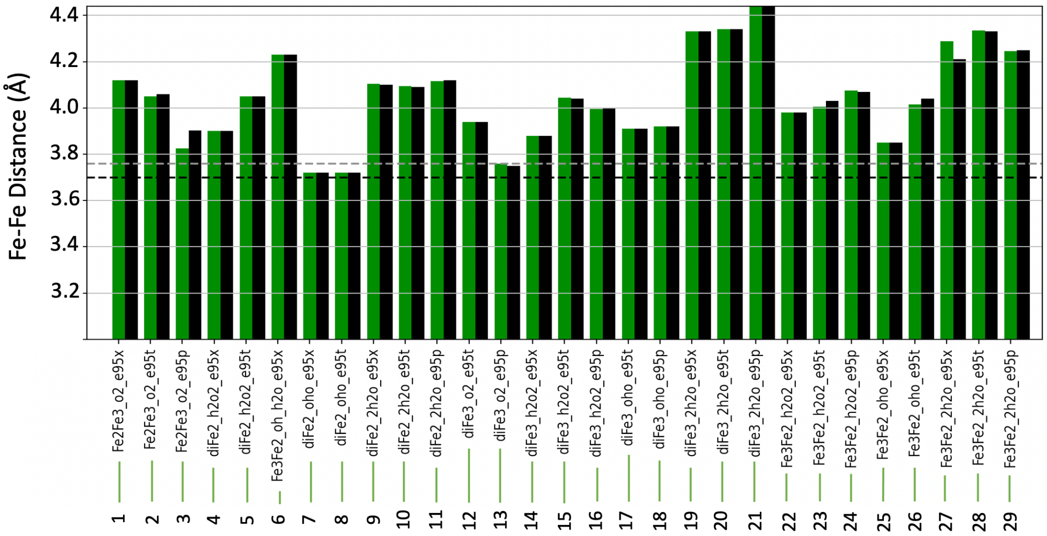

Figure S2: Magnetic exchange coupling (J, cm^{-1}) as a function of the **Fe–Fe distance** (Å) for QM/MM-optimized diiron models with Fe–Fe separations below 4.0 Å. The yellow-shaded area marks the regime of very weak coupling ($|J|<15$ cm^{-1}), corresponding to an effectively diamagnetic diiron site.

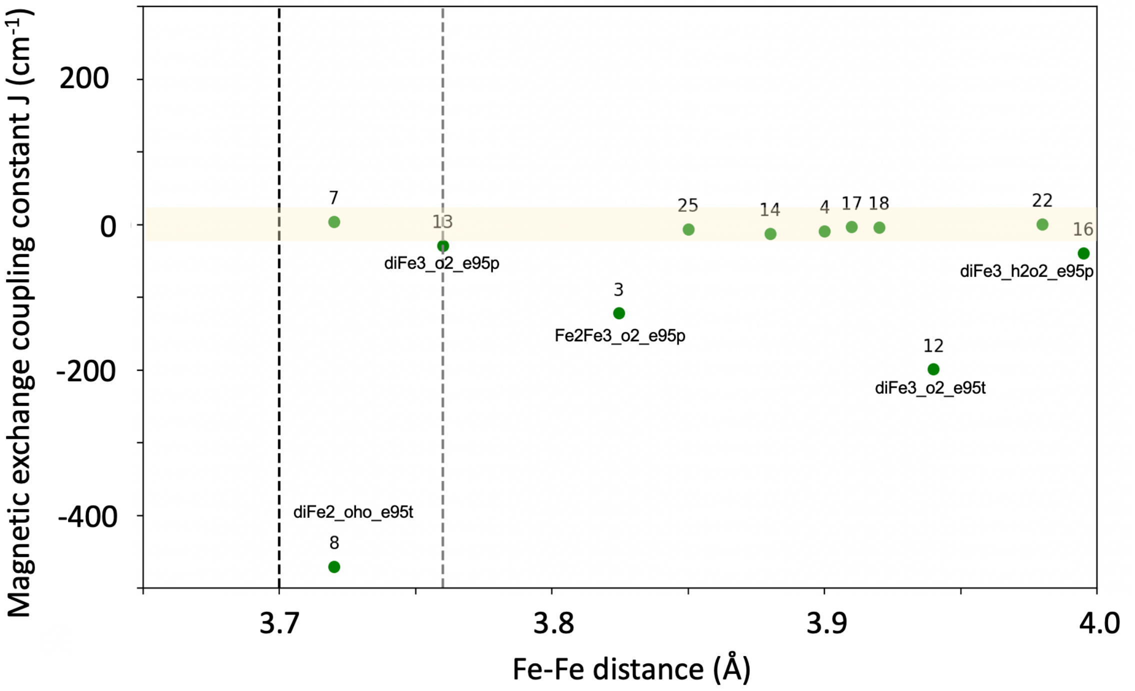

Figure S3: Comparison of high-spin structural models of active site 1 of diferrous SulE (left panels) with the corresponding mixed-valence Fe(III)–Fe(II) models (right panels), shown for both the deprotonated (anionic) Glu95 (upper panels) and protonated Glu95 (lower panels). The RMSD between the diferrous and mixed-valence models, calculated over all heavy atoms in the QM region, is 0.20 Å for both protonation states of Glu95.

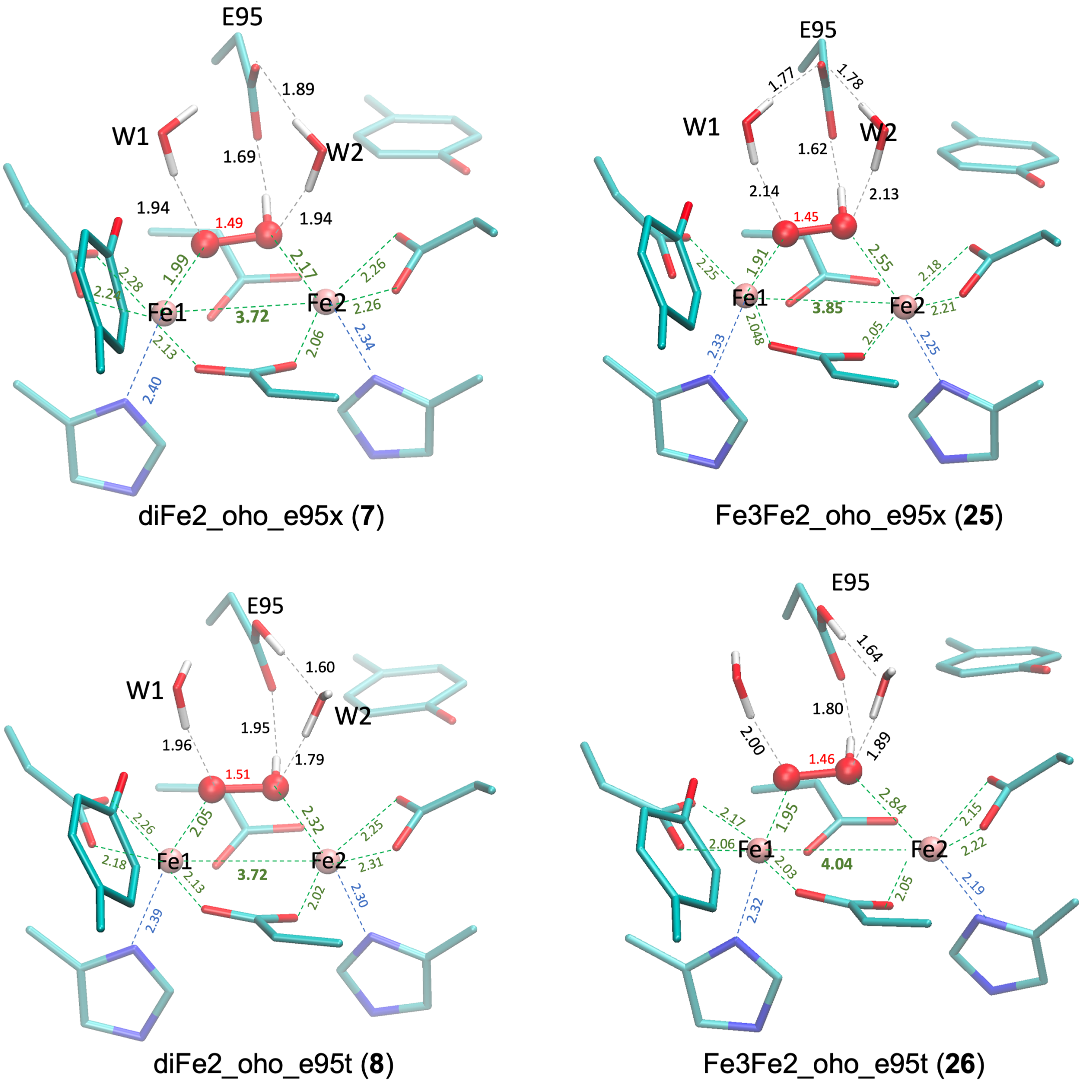

Supplement: Supplementary file 1 — Supplementary Material [file CPHC-27-e202500772-s001.pdf]
